# The Impact of Coenzyme Q10 on Cognitive Dysfunction, Antioxidant Defense, Cholinergic Activity, and Hippocampal Neuronal Damage in Monosodium Glutamate-Induced Obesity

**DOI:** 10.5812/ijpr-157068

**Published:** 2025-01-05

**Authors:** Zahra Erfanmanesh, Mohammad Amin Edalatmanesh, Mokhtar Mokhtari

**Affiliations:** 1 Department of Biology, Shiraz Branch, Islamic Azad University, Shiraz, Iran; 2 Department of Biology, Kazeroon Branch, Islamic Azad University, Kazeroon, Iran

**Keywords:** Ubiquinone, Monosodium Glutamate, Hippocampus, Obesity, Rat

## Abstract

**Background:**

Obesity, a rising global health issue, is linked to numerous disorders, including cognitive impairment.

**Objectives:**

This study investigates the effects of coenzyme Q10 (Co-Q10) on cognitive performance, antioxidant defense, cholinergic activity, and hippocampal neuron damage in rats rendered obese by monosodium glutamate (MSG) exposure.

**Methods:**

Forty-eight neonatal male Wistar rats were randomly assigned to one of four groups: Control, MSG, MSG + Q10-10, and MSG + Q10-20. Monosodium glutamate (4 g/kg BW) was administered subcutaneously into the cervical region from postnatal day (PND) 2 to PND 10. Coenzyme Q10 (10 mg/kg BW and 20 mg/kg BW) was administered intraperitoneally from PND 30 to PND 42. At the end of the treatment period, working memory and avoidance learning tests were conducted. Anthropometric data were collected, followed by evaluations of hippocampal catalase (CAT), superoxide dismutase (SOD), acetylcholinesterase (AChE), glutathione peroxidase (GPx), and malondialdehyde (MDA) levels. The density of apoptotic/dark neurons (DN) in the CA₁ and CA₃ regions of the hippocampus was also assessed.

**Results:**

Monosodium glutamate treatment increased Body Mass Index (BMI) and Lee Index, impaired working memory and avoidance learning, and reduced CAT, SOD, and GPx activities. Additionally, MSG exposure led to elevated MDA levels, increased AChE activity, and higher DN density in the CA₁ and CA₃ hippocampal regions. Treatment with Co-Q10 resulted in a decrease in BMI, enhanced memory and learning, noteworthy increases in CAT, SOD, and GPx activities in the hippocampus, and reductions in MDA levels, AChE activity, and DN density in the CA₁ and CA₃ regions.

**Conclusions:**

Coenzyme Q10 mitigates hippocampal neuronal damage and improves cognitive function in MSG-induced obesity, primarily through its antioxidant and AChE inhibitory properties.

## 1. Background

The World Health Organization (WHO) classifies obesity as a global epidemic and one of the most noteworthy public health challenges today. According to WHO, obesity and overweight contribute to 2.8 million deaths annually, with global prevalence more than doubling since 1980 (1). Obesity noteworthyly elevates the risk of various cancers, respiratory diseases, arthritis, hypertension, dyslipidemia, cardiovascular disease, and type 2 diabetes (2). Furthermore, childhood obesity has been linked to a higher likelihood of developing cognitive disorders and dementia later in life (3).

Monosodium glutamate (MSG)-induced obesity, achieved through high-dose administration in the early postnatal period in rodents, leads to necrosis in hypothalamic structures, resulting in behavioral, cognitive, and motor dysfunction (4). Neonatal administration of MSG specifically destroys the ventromedial hypothalamic nucleus (VMH), leading to stunted growth and obesity (5).

Monosodium glutamate (E621) is a widely used flavor enhancer and legally approved food additive in many countries, particularly in Asia. Although its use is unrestricted in many regions, excessive consumption of MSG has toxic effects and increases the risk of obesity (6). In addition, MSG has been shown to cause structural brain changes, neurotoxicity, and oxidative stress (7). Overconsumption of MSG has been linked to neurodegenerative damage, Parkinson's disease (PD), epilepsy (E), and the stimulation of norepinephrine and dopamine release in various brain regions (8). Monosodium glutamate is particularly damaging to hypothalamic and hippocampal neurons, and neuronal damage and necrosis are monitored in several brain areas, including the hippocampus, following high doses of MSG due to increased glutamate levels (9).

The hippocampus plays a critical role in memory formation and behavioral regulation. Monosodium glutamate-induced damage to the hippocampus is associated with the formation of reactive oxygen species (ROS) and the impairment of antioxidant defenses. Since hippocampal intracellular and synaptic events continue to develop after birth, neonatal MSG exposure disrupts these critical processes (10).

The molecular mechanisms behind MSG-induced learning and memory impairments are thought to involve neurotransmitter imbalances and altered activity of enzymes such as acetylcholinesterase (AChE) and dopamine β-hydroxylase, which are involved in neurotransmitter metabolism (11). Research indicates that AChE is closely related to brain development, learning, memory, and neuronal damage. Increased AChE activity leads to rapid degradation of acetylcholine, resulting in diminished acetylcholine receptor stimulation and impaired cognitive function (12).

While many drugs previously used to manage obesity have been discontinued due to severe side effects, natural antioxidant compounds have gained acceptance as a safer therapeutic alternative (13). Coenzyme Q10 (Co-Q10), a fat-soluble benzoquinone found in all cells of the body, serves multiple functions despite its limited dietary availability (14). As an antioxidant, Co-Q10 prevents mitochondrial pore opening, protects cells from destruction, and exhibits anti-apoptotic properties (15). Brain levels of Co-Q10 decrease with age, making it a potential therapeutic agent for age-related neurodegenerative disorders (16). Coenzyme Q10 has been studied for its role in improving neurological disorders, including Alzheimer's disease AD, PD, depression (MDD), E, multiple sclerosis (MS), brain injuries (TBI), spinocerebellar ataxia (SCA), and Huntington's chorea (HD) (17).

## 2. Objectives

This study aims to investigate the effects of Co-Q10 on working and avoidance memory, oxidative stress markers, AChE activity, and apoptotic/dark neuron density in the hippocampus of MSG-induced obese rats.

## 3. Methods

### 3.1. Animal Treatment and Experimental Design

In this experimental study, 48 male neonatal Wistar rats were used. Twenty-five pregnant Wistar rats with known gestational day zero were obtained from the Razi Serum Institute and housed under standard conditions in the animal laboratory at Islamic Azad University, Shiraz. Each pregnant rat was housed individually in standard polycarbonate cages (manufactured by Razi Rad, Iran) under controlled conditions, with a temperature of 25 ± 1°C, 45 ± 5% relative humidity, and a 12/12-hour light/dark cycle (lights on at 7:00 AM). Standard rat chow and purified water were provided ad libitum. All experimental procedures followed international ethical guidelines and were approved by the Animal Ethics Committee of Islamic Azad University, Shiraz (ethics code: IR.IAU.SHIRAZ.REC.1399.009). Every effort was made to minimize animal use and reduce pain or discomfort. All dams delivered naturally, and neonate numbers and birth weights were recorded. The neonates stayed with their mothers until postnatal day (PND) 21, after which they were weaned and housed individually with standard food.

The neonates were randomly assigned (2 to 3 male offspring from each mother) to one of four groups (n = 12 per group): Control, MSG, MSG + Q10-10, and MSG + Q10-20. The control group received no treatment. In the MSG group, MSG (Sigma, Germany) was administered subcutaneously at a dose of 4 g/kg body weight from PND2 to PND10 in the cervical region (18). From PND30 to PND42, animals in this group received the Co-Q10 vehicle (sesame oil) intraperitoneally (I.P.) daily. In the MSG + Q10-10 and MSG + Q10-20 groups, in addition to MSG administration during the neonatal period, Co-Q10 (Sigma, Germany) was administered I.P. at doses of 10 mg/kg and 20 mg/kg body weight (19), respectively, from PND30 to PND42, along with the sesame oil vehicle. No mortality was monitored during the treatment period.

Anthropometric parameters, including naso-anal length and weight changes relative to birth weight, were measured on PND50. Body Mass Index (BMI) was calculated by dividing body weight (g) by the square of body length (cm²), and the Lee Index, an indicator of obesity, was determined by dividing the cube root of body weight (g) by naso-anal length (cm) × 1000 (20).

### 3.2. Behavioral Tests Battery

#### 3.2.1. Working Memory

At the end of the treatment period, on PND43, spatial working memory was assessed using the Y-maze for all groups (n = 12). The Y-maze consists of three identical arms, each measuring 15 ×30 × 40 cm, connected to a central area. At the start of the test, each animal was gently placed into one of the three arms without inducing stress, and its movements were monitored for 5 minutes. The number of entries into each arm was recorded, with an entry being defined as the placement of the animal's hind limb into an arm.

Alternation behavior, a measure of working memory, was defined as consecutive entries into all three arms in overlapping triplet sets. The percentage of alternation behavior, representing working memory performance, was calculated as the number of successful alternations divided by the total number of arm entries minus two, then multiplied by 100. A successful alternation was defined as three consecutive entries into all three arms in sequence (21).

#### 3.2.2. Passive Avoidance Memory

On PND45, passive avoidance memory was evaluated in all groups (n = 12) using a shuttle box. The shuttle box consists of two compartments, one illuminated and one dark, with identical dimensions (27 × 14.5 × 14 cm) separated by a guillotine door. The floor of the dark compartment is equipped with steel rods (2 mm in diameter) connected to an electrical circuit. When activated, an electric current with specified duration, intensity, and frequency passes through the floor. The test is based on the natural tendency of rodents to avoid light and the aversive stimulus (electric shock) in the dark compartment. The test is divided into three main phases:

##### 3.2.2.1. Habituation

Each animal was placed in the illuminated compartment for one minute to acclimate to the environment. After 30 seconds, the guillotine door was opened, allowing the animal to move freely into the dark compartment.

##### 3.2.2.2. Acquisition

This phase took place 24 hours after habituation. The animal was placed in the illuminated compartment, and after 30 seconds, the guillotine door was opened, allowing it to enter the dark compartment. Upon entry, the guillotine door was closed, and an electric shock (2 mA, 2 seconds, 50 Hz) was delivered through the floor. The animal was removed 20 seconds after the shock and returned to its cage.

##### 3.2.2.3. Retention

Retention tests were conducted 24 and 48 hours after the acquisition phase. During these tests, the animal was placed in the illuminated compartment, and after 30 seconds, the guillotine door was opened. The latency to enter the dark compartment (LDR) and the total time spent in the dark compartment (TDR) were recorded (22). No electric shocks were given during the retention tests. The cutoff time for the test was 300 seconds, and a longer delay in entering the dark compartment (LDR) was interpreted as successful memory retention.

### 3.3. Biochemical Studies

#### 3.3.1. Oxidative Stress Parameters

On PND50, following the completion of behavioral tests, a subset of animals from each group (n = 8) was deeply anesthetized using a mixture of ketamine (50 mg/kg) and xylazine (5 mg/kg), after which the animals were immediately decapitated using a specialized rodent guillotine. The entire brain was rapidly removed and placed on ice. Under a stereoscope (Olympus, Japan), the hippocampus was carefully dissected from the brain. The tissue was washed with normal saline and Tris buffer (Sigma, Germany) and then homogenized using a homogenizer (IKA, Germany) for 10 minutes at 5000 rpm. The homogenized solution was subsequently centrifuged in a refrigerated centrifuge (Hermle, Germany), and 0.5 mM phenylmethylsulfonyl fluoride (PMSF; Sigma-Aldrich, Germany) was added as a protease inhibitor (23). The supernatant obtained from the centrifugation process was used to assess oxidative stress markers.

Tissue levels of catalase (CAT), superoxide dismutase (SOD), and glutathione peroxidase (GPx) were measured using ELISA kits (Kia Zist, Iran) and an ELISA reader (Stat Fax, USA). Malondialdehyde (MDA) levels in the tissue were determined via spectrophotometry by measuring the reaction of MDA with thiobarbituric acid (Merck, Germany), with absorbance readings at 535 nm compared against a standard curve to quantify MDA concentration in the tissue samples.

#### 3.3.2. Cholinergic Activity

Acetylcholinesterase activity in hippocampal tissue was measured via ACh hydrolysis using the Ellman method (24). A volume of 0.4 mL of the supernatant from hippocampal tissue homogenization was mixed with 2.6 mL of phosphate buffer (0.1 M, pH 7.4). Afterward, 0.1 mL of DTNB was added to the mixture, followed by 0.1 mL of acetylthiocholine iodide. Absorbance was measured at 412 nm with a spectrophotometer, and the change in absorbance was monitored over 2 minutes.

Acetylcholinesterase activity was determined by the increase in color resulting from the reaction between thiocholine and DTNB. The rate of absorbance change per minute was calculated, and AChE activity was expressed as micromoles of substrate hydrolyzed per minute per milligram of protein.

### 3.4. Histological Studies

To assess the density of dark neurons (DNs) in the hippocampus, a subset of animals from each group (n = 4) was anesthetized with a mixture of ketamine (50 mg/kg) and xylazine (5 mg/kg) on PND50, and immediate cardiac perfusion was performed. Following the fixation of brain tissue through cardiac perfusion, the brain was carefully removed from the skull and processed for histological studies using an autotechnicon. Parrafin blocks were prepared, and frontal sections of the hippocampus were made according to the Paxinos and Watson atlas. To assess the density of DNs, 1% toluidine blue staining was used (25). Imaging was performed using a light microscope (Olympus-BH2, Japan) with a 40x objective lens (total magnification of 400x).

After random sectioning, the density of DNs in the CA₁ and CA₃ regions of the hippocampus was measured using the disector method. Briefly, serial sections with a specified interval were prepared from the entire hippocampal region. In each section, cells were counted within a reference framework. The neuronal density was then calculated using the formula.


$$NA=\frac{\sum Q}{\sum P\times AH}$$

Where NA is the neuronal density, ∑Q is the total number of counted cells in a sample, ∑P is the number of sampling occurrences in a sample, A is the area of the sampling framework, and H is the distance between two consecutive sections or the thickness of each section (26). A minimum of 10 slides from each hippocampal sample (40 slides per group) were counted to evaluate the DN density.

### 3.5. Statistical Analysis

Statistical analysis of the data obtained from the various groups was conducted using SPSS software version 26. A one-way ANOVA followed by Tukey's post hoc test was performed to determine the presence of noteworthy divergences between the groups. Statistically noteworthy divergences were considered at P < 0.05.

## 4. Results

### 4.1. Changes in Body Weight, Body Length, Body Mass Index, and Lee Index

The study groups monitored Noteworthy divergences in body weight, BMI, and Lee Index (Table 1). A noteworthy increase in body weight was monitored in the MSG group in relation to the control group. The MSG group showed noteworthy increases in body weight (P < 0.05), BMI (P < 0.01), and Lee Index (P < 0.05) and a noteworthy decrease in body length (P < 0.05) in relation to the control group. In contrast, the groups receiving Co-Q10 showed no noteworthy divergences in the parameters in relation to the control group. In relation to the MSG group, the MSG + Q10-10 group showed a noteworthy increase in body length (P < 0.05) and a noteworthy decrease in BMI (P < 0.01). Moreover, the MSG + Q10-20 group exhibited a noteworthy reduction in body weight (P < 0.05) and BMI (P < 0.01) and a noteworthy increase in body length (P < 0.05) in relation to the MSG group.

**Table 1.** Mean ± Standard Deviation of Body Weight, Body Length, Body Mass Index, and Lee Index ^a^

Parameters	Groups			
	Control	MSG	MSG + Q10-10	MSG + Q10-20
**Body weight (g)**	228.42 ± 19.6	260.09 ± 21.2 ^*^	245.13 ± 15.4	232.36 ± 16.7 ^+^
**Body length (cm)**	20.3 ± 0.9	19.1 ± 0.6 ^*^	20.5 ± 0.8 ^+^	20.0 ± 0.5 ^+^
**BMI (g/cm** ^**2**^ **)**	0.55 ± 0.05	0.71 ± 0.08 ^**^	0.58 ± 0.07 ^++^	0.57 ± 0.07 ^++^
**Lee’s Index (g/cm)**	0.30 ± 0.01	0.33 ± 0.01 ^*^	0.31 ± 0.02	0.31 ± 0.01

Abbreviations: BMI, Body Mass Index; MSG, monosodium glutamate.

^a^ Noteworthy divergences were monitored in the above parameters in relation to the control group (** P < 0.01, * P < 0.05). Furthermore, noteworthy divergences were monitored in some parameters between the MSG + Q10-10 and MSG + Q10-20 groups in relation to the MSG group (++ P < 0.01, + P < 0.05).

### 4.2. Percentage of Alternation Behavior

**Figure 1.**
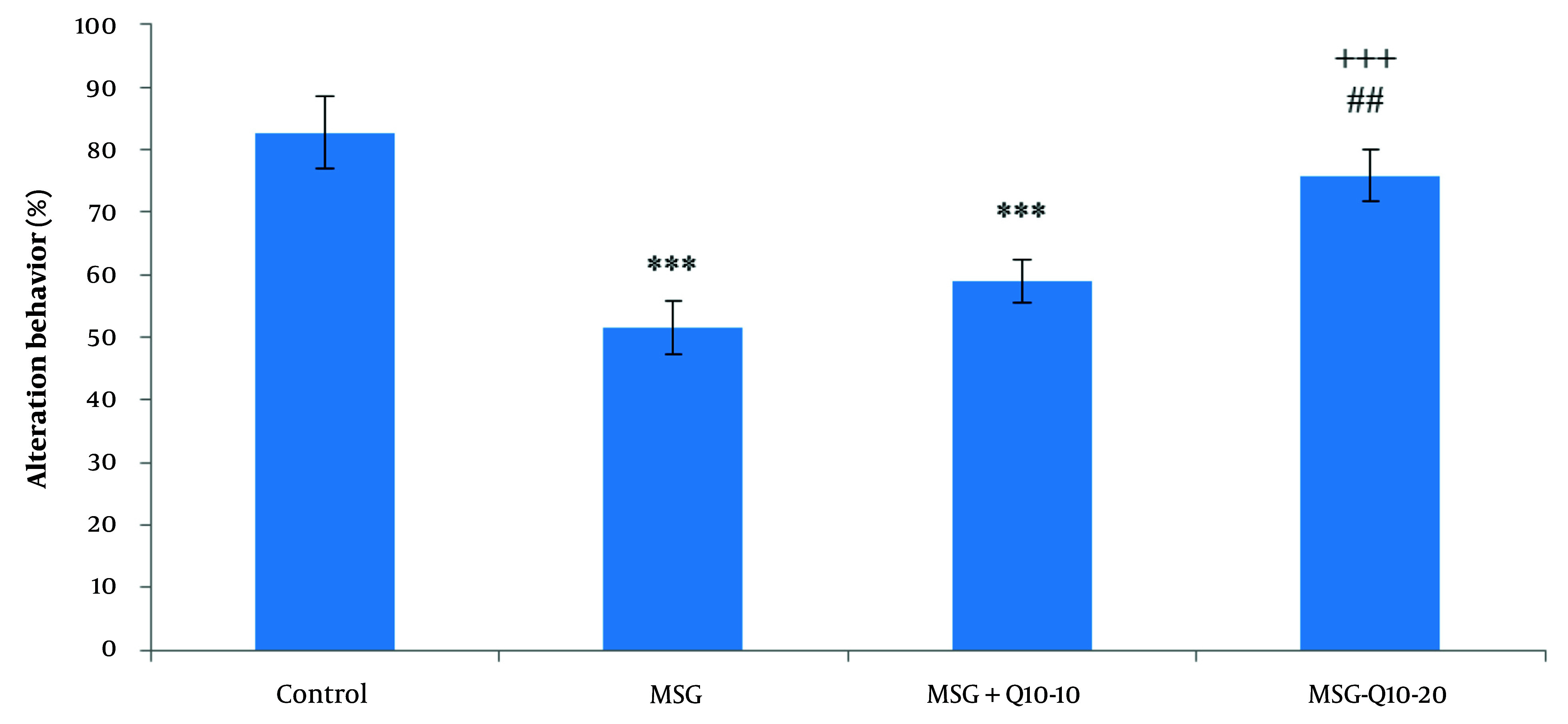
Comparison of mean ± standard deviation of the percentage of alternation behavior among the study groups. Noteworthy divergences were monitored between the control group and the monosodium glutamate (MSG) and MSG + Q10-10 groups (*** P < 0.001). In relation to the MSG group, the MSG + Q10-20 group showed a noteworthy increase (+++ P < 0.001). A noteworthy divergence was also monitored between the MSG + Q10-10 and MSG + Q10-20 groups (## P < 0.01).

Statistical analysis of alternation behavior percentage using one-way ANOVA and Tukey's post hoc test revealed noteworthy divergences among the study groups (Figure 1). Specifically, the percentage of alternation behavior (working memory) was noteworthyly reduced in the MSG and MSG + Q10-10 groups in relation to the control group (P < 0.001). However, the MSG + Q10-20 group showed a noteworthy increase in the percentage of alternation behavior in relation to the MSG group (P < 0.001). A noteworthy divergence was also monitored between the MSG + Q10-10 and MSG + Q10-20 groups (P < 0.01).

### 4.3. Passive Avoidance Memory

**Figure 2.**
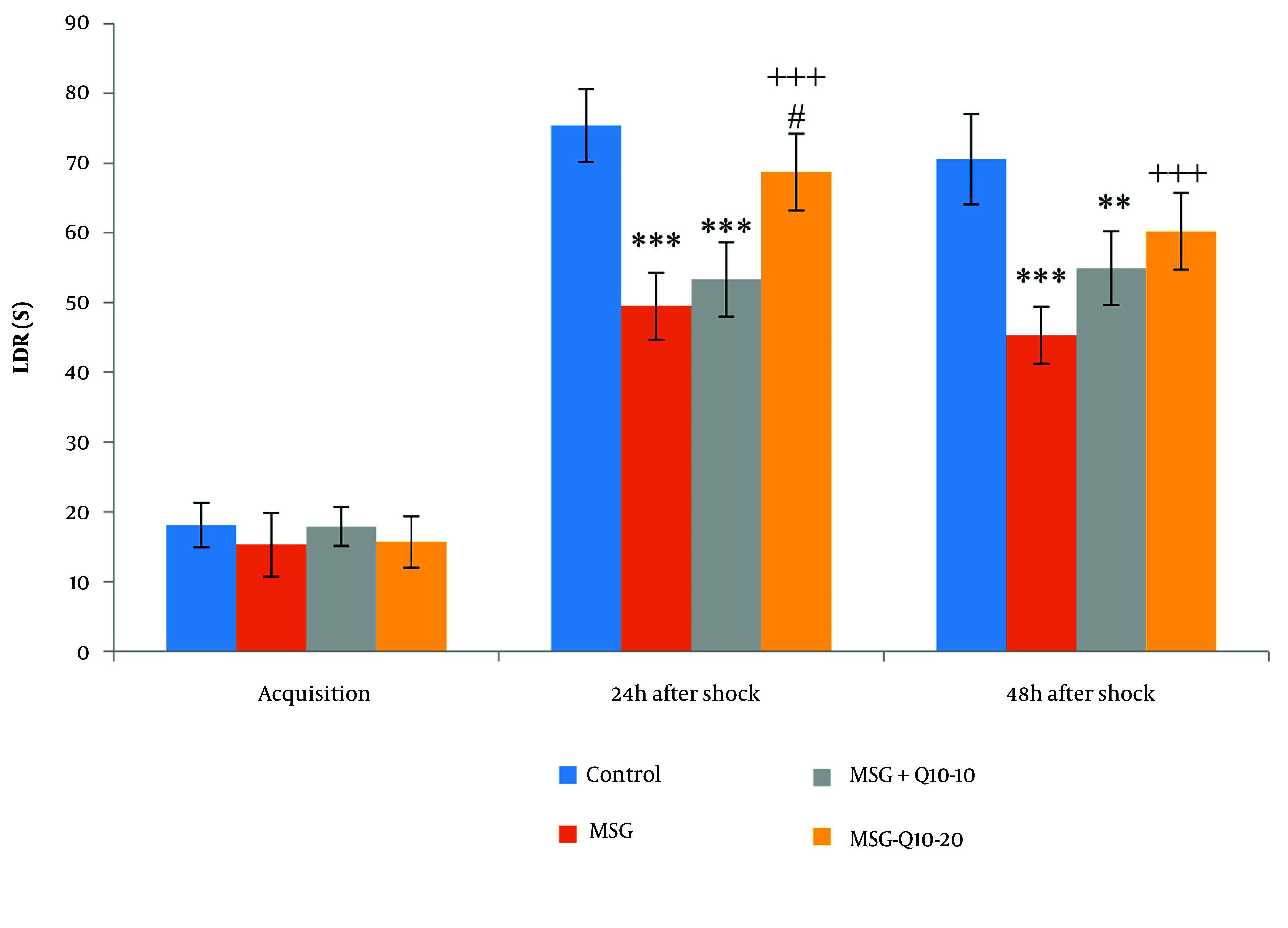
Comparison of mean ± standard deviation of LDR. During the acquisition phase, no noteworthy divergences were monitored between the groups. However, 24 and 48 hours post-shock, a noteworthy divergence was monitored between the control group and the monosodium glutamate (MSG) and MSG + Q10-10 groups (*** P < 0.001 and ** P < 0.01, respectively). In relation to the MSG group, the MSG + Q10-20 group showed a noteworthy increase at both time intervals after the shock (+++ P < 0.001). Furthermore, 24 hours after the shock, a noteworthy divergence was noted between the MSG + Q10-10 and MSG + Q10-20 groups (# P < 0.05).

No noteworthy divergences in latency to enter the dark room (LDR) were monitored among the study groups during the acquisition phase (Figure 2). However, noteworthy divergences in LDR were noted at 24 and 48 hours after shock induction. At 24 hours post-shock, the MSG and MSG + Q10-10 groups exhibited a noteworthy decrease in LDR in relation to the control group (P < 0.001). Similarly, at 48 hours post-shock, both the MSG (P < 0.001) and MSG + Q10-10 (P < 0.01) groups showed a noteworthy reduction in LDR in relation to the control group. In contrast, the MSG + Q10-20 group demonstrated a noteworthy increase in LDR in relation to the MSG group at both 24 and 48 hours post-shock (P < 0.001). Additionally, the MSG + Q10-20 group showed a noteworthy increase in LDR in relation to the MSG + Q10-10 group at 24 hours post-shock (P < 0.05).

**Figure 3.**
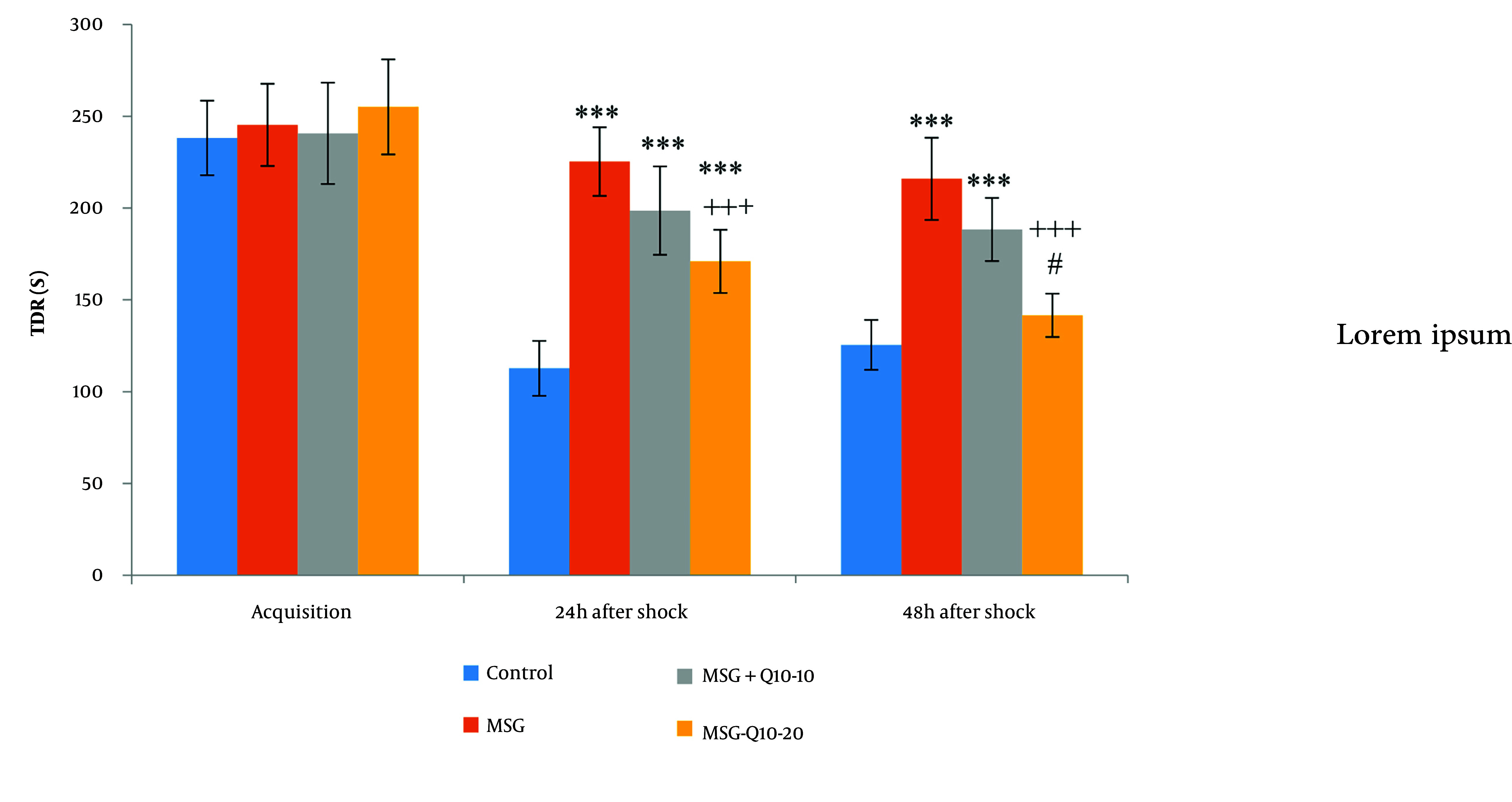
Comparison of mean ± standard deviation of TDR. No noteworthy divergences were monitored during the training phase among the study groups. However, in relation to the control group, noteworthy divergences were monitored in the MSG, MSG + Q10-10, and MSG + Q10-20 groups 24 hours post-shock and in the MSG and MSG + Q10-10 groups 48 hours post-shock (*** P < 0.001). The MSG + Q10-20 group showed a noteworthy decrease in TDR in relation to the MSG group at both time intervals post-shock (+++ P < 0.001). A noteworthy divergence was monitored between the MSG + Q10-10 and MSG + Q10-20 groups 48 hours after the shock (# P < 0.05).

The mean time spent in the darkroom (TDR) during the acquisition phase showed no noteworthy divergences among the groups (Figure 3). However, noteworthy divergences in TDR were monitored at 24 and 48 hours after shock. At 24 hours post-shock, the MSG, MSG + Q10-10, and MSG + Q10-20 groups demonstrated a noteworthy increase in TDR in relation to the control group (P < 0.001). Similarly, at 48 hours post-shock, the MSG and MSG + Q10-10 (P < 0.001) groups showed a noteworthy increase in TDR in relation to the control group. Conversely, the MSG + Q10-20 group showed a noteworthy decrease in TDR in relation to the MSG group at both 24 and 48 hours post-shock (P < 0.001). A noteworthy divergence in TDR was also monitored between the MSG + Q10-10 and MSG + Q10-20 groups at 48 hours post-shock (P < 0.05).

During the acquisition phase, no noteworthy divergences were monitored in the TDR among the study groups (Figure 3). However, 24 and 48 hours after the shock, the TDR noteworthyly differed across the study groups. In relation to the control group, 24 hours after the shock, the MSG, MSG + Q10-10, and MSG + Q10-20 groups showed a noteworthy increase (P < 0.001). Additionally, 48 hours after the shock, the MSG and MSG + Q10-10 groups exhibited a noteworthy increase in relation to the control group (P < 0.001 and P < 0.01, respectively). In contrast, the MSG + Q10-20 group showed a noteworthy decrease in TDR in relation to the MSG group at 24 and 48 hours post-shock (P < 0.001). A noteworthy divergence was also monitored between the MSG + Q10-10 and MSG + Q10-20 groups 48 hours after the shock (P < 0.05).

### 4.4. Oxidative Stress Factors

The evaluation of CAT enzyme activity in the hippocampus showed noteworthy divergences among the study groups (Table 2). The MSG, MSG + Q10-10, and MSG + Q10-20 groups showed a noteworthy decrease in relation to the control group (P < 0.001). Furthermore, in relation to the MSG group, the MSG + Q10-10 and MSG + Q10-20 groups exhibited a noteworthy increase (P < 0.01 and P < 0.001, respectively).

The analysis of hippocampal SOD activity also revealed noteworthy divergences among the study groups (Table 2). Superoxide dismutase activity in the MSG and MSG + Q10-10 groups was noteworthyly decreased in relation to the control group (P < 0.01 and P < 0.001, respectively). Conversely, the MSG + Q10-20 group showed a noteworthy increase in SOD activity in relation to the MSG group (P < 0.001). Moreover, a noteworthy divergence was monitored between the MSG + Q10-10 and MSG + Q10-20 groups (P < 0.05).

**Table 2.** Mean ± Standard Deviation of Catalase, Superoxide dismutase, Glutathione Peroxidase Enzymatic Activities, and Monosodium Glutamate Content in the Hippocampus ^a^

Groups	Parameters			
	CAT (mIU/mL)	SOD (pg/mL)	GPx (mIU/mL)	MDA (ng/mL)
**Control**	107.22 ± 7.6	73.49 ± 6.1	105.19 ± 6.1	32.07 ± 3.1
**MSG**	54.31 ± 6.5 ^***^	47.23 ± 4.8 ^***^	41.83 ± 4.7 ^***^	56.62 ± 4.7 ^***^
**MSG + Q10-10**	73.04 ± 6.9 ^***, ++^	52.31 ± 5.7 ^**^	63.95 ± 6.5 ^***, ++^	48.31 ± 4.5 ^**^
**MSG + Q10-20**	79.51 ± 5.4 ^***, +++^	67.82 ± 6.5 ^+++, #^	91.70 ± 5.8 ^*, +++, ###^	38.94 ± 3.7 ^+++, ##^

Abbreviations: CAT, catalase; SOD, superoxide dismutase; GPx, glutathione peroxidase; MDA, malondialdehyde; MSG, monosodium glutamate

^a^ In relation to the control group, the MSG, MSG + Q10-10, and MSG + Q10-20 groups showed noteworthy divergences in the above parameters (*** P < 0.001, ** P < 0.01, and * P < 0.05). Additionally, in relation to the MSG group, noteworthy divergences were monitored in the MSG + Q10-10 and MSG + Q10-20 groups (+++ P < 0.001 and ++ P < 0.01). Moreover, a noteworthy divergence was monitored between the MSG + Q10-10 and MSG + Q10-20 groups (### P < 0.001, ## P < 0.01, and # P < 0.05).

Hippocampal GPx levels also noteworthyly differed across the groups (Table 2). In relation to the control group, the MSG, MSG + Q10-10, and MSG + Q10-20 groups showed a noteworthy decrease (P < 0.001, P < 0.001, and P < 0.05, respectively). In relation to the MSG group, the MSG + Q10-10 and MSG + Q10-20 groups have demonstrated a noteworthy increase (P < 0.01 and P < 0.001, respectively). Furthermore, a noteworthy divergence was monitored between the MSG + Q10-10 and MSG + Q10-20 groups (P < 0.001).

The analysis of hippocampal MDA content indicated noteworthy divergences among the study groups (Table 2). Specifically, a noteworthy increase was monitored in the MSG and MSG + Q10-10 groups in relation to the control group (P < 0.001 and P < 0.01, respectively). In relation to the MSG group, the MSG + Q10-20 group exhibited a noteworthy decrease (P < 0.001). A noteworthy divergence was also monitored between the MSG + Q10-10 and MSG + Q 10 - 20 groups (P < 0.01).

### 4.5. Cholinergic Activity

**Figure 4.**
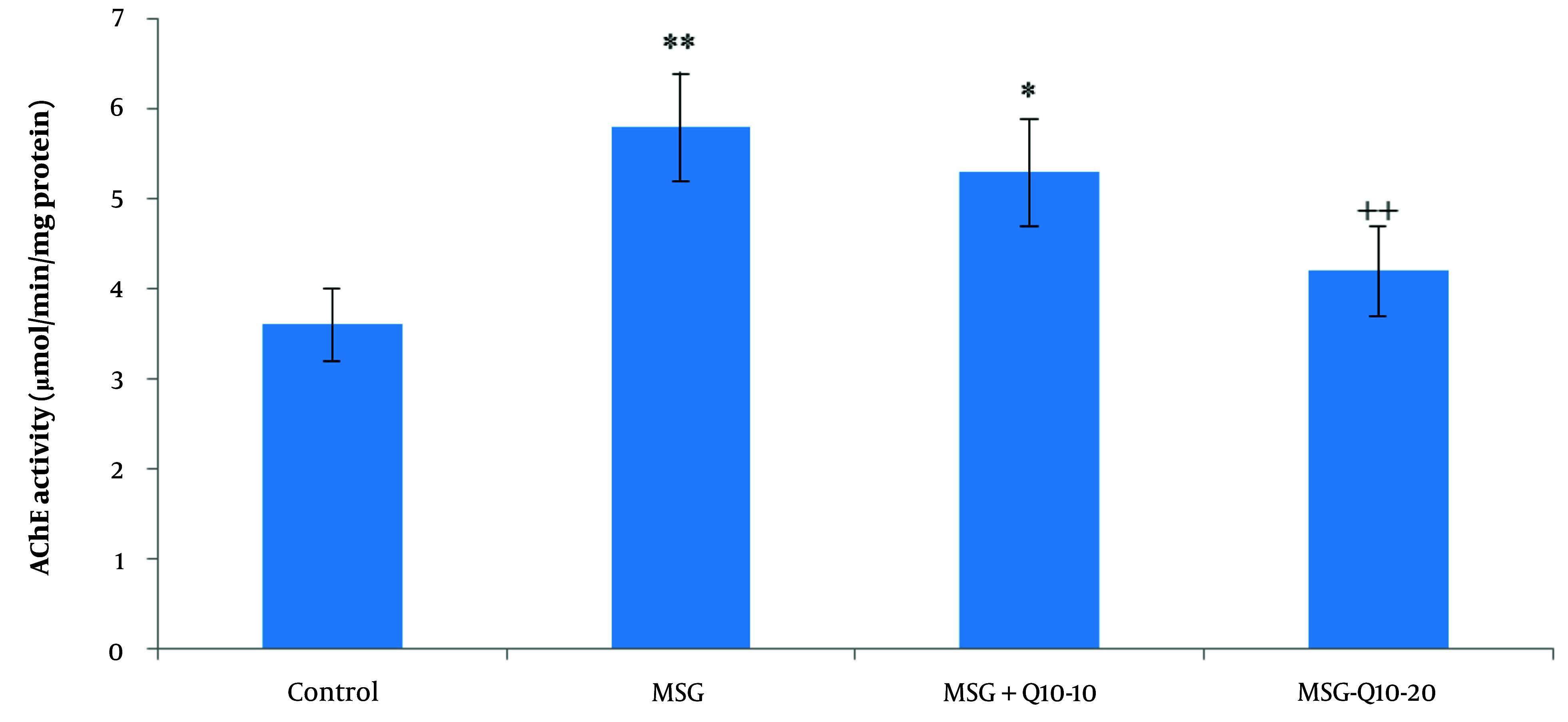
Comparison of mean ± standard deviation of hippocampal acetylcholinesterase (AChE) activity across study groups. The results indicate a noteworthy divergence between the control group and the monosodium glutamate (MSG) and MSG + Q10-10 groups (** P < 0.01 and * P < 0.05, respectively). The MSG + Q10-20 group demonstrated a noteworthy decrease in AChE activity in relation to the MSG group (++ P < 0.01).

The data analysis revealed a noteworthy divergence in hippocampal AChE activity among the study groups (Figure 4). Acetylcholinesterase activity in the MSG and MSG + Q10-10 groups was noteworthyly increased in relation to the control group (P < 0.01 and P < 0.05, respectively). Additionally, in relation to the MSG group, the MSG + Q10-20 group showed a noteworthy decrease in hippocampal AChE activity (P < 0.01).

### 4.6. Density of DNs

**Figure 5.**
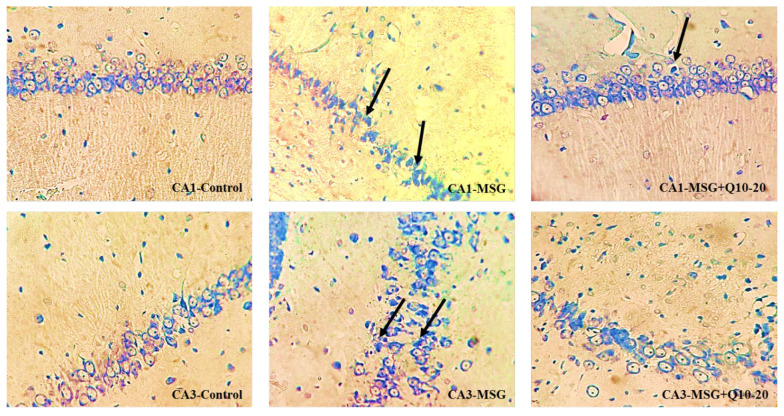
Photomicrographs of the CA₁ and CA₃ regions of the hippocampus across study groups. An increased density of dark neurons (DNs) is monitored in the monosodium glutamate (MSG) group in relation to the control group. Conversely, a lower density of DNs is noticeable in the MSG + Q10-20 group. Toluidine blue staining. Black arrows indicate DN. Magnification: 40X.

**Figure 6.**
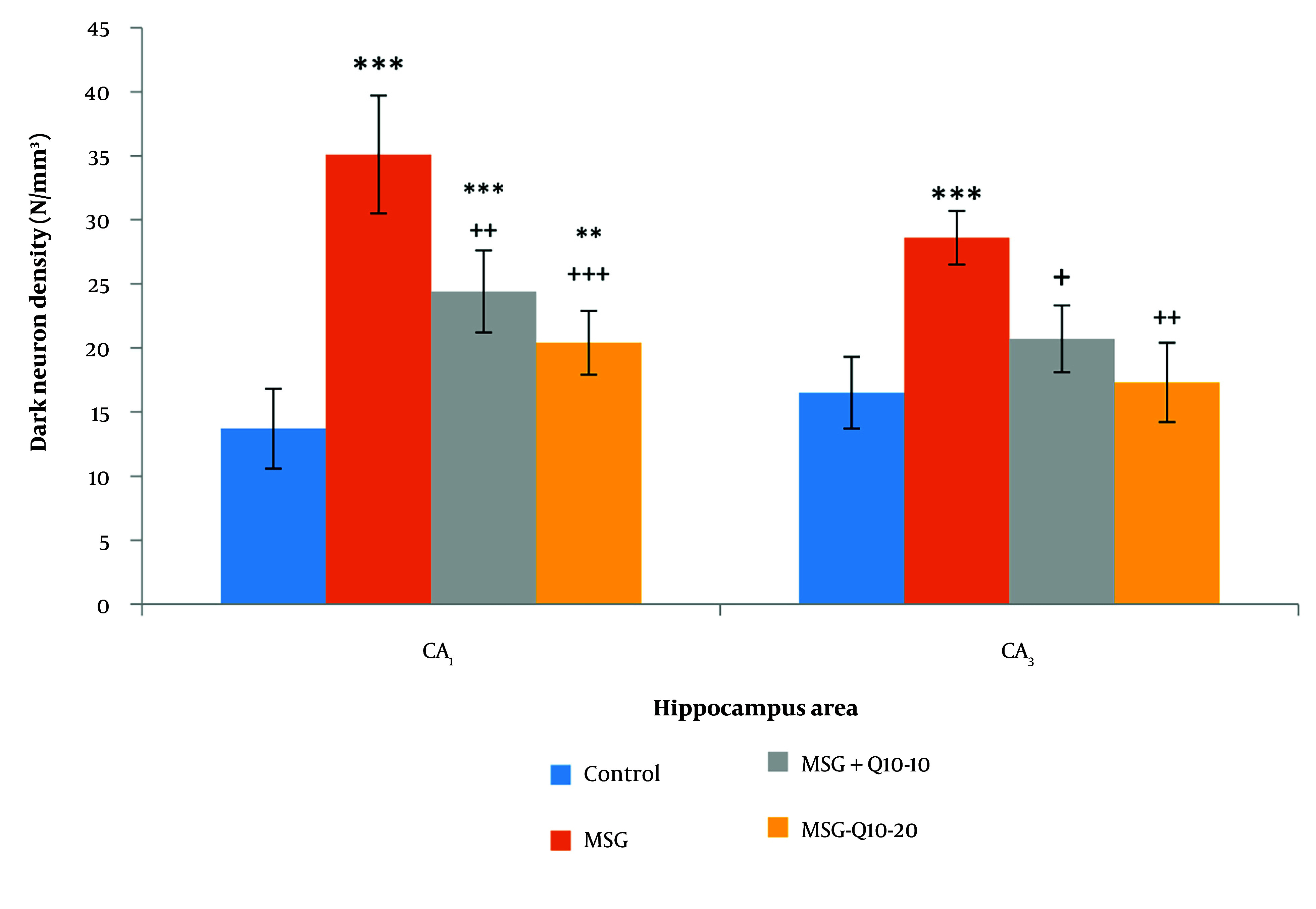
Comparison of mean ± standard deviation of dark neurons (DN) density in the and CA₃ regions of the hippocampus across different groups. Noteworthy divergences were monitored between the control group and the monosodium glutamate (MSG), MSG + Q10-10, and MSG + Q10-20 groups in the region and with the MSG group in the CA₃ region (*** P < 0.001 and ** P < 0.01, respectively). Additionally, in relation to the MSG group, the MSG + Q10-10 and MSG + Q10-20 groups demonstrated a noteworthy decrease in dark neuron density in both regions (+++ P < 0.001, ++ P < 0.01, and + P < 0.05, respectively).

DNs were monitored in various brain regions using Toluidine Blue Staining. This study examined the density of DNs in the CA₁ and CA₃ regions of the hippocampus across different groups (Figure 5). The data analysis showed a noteworthy divergence between the control and MSG groups in the CA₁ and CA₃ regions (P < 0.001). In the CA₁ region, the MSG + Q10-10 and MSG + Q10-20 groups showed a noteworthy increase in dark neuron density in relation to the control group (P < 0.001 and P < 0.01, respectively). In relation to the MSG group, the MSG + Q10-10 and MSG + Q10-20 groups exhibited a noteworthy decrease in dark neuron density in the CA₁ region (P < 0.01 and P < 0.001, respectively) and in the CA₃ region (P < 0.05 and P < 0.01, respectively) (Figure 6). 

## 5. Discussion

Obesity is regarded as the most prevalent health issue and nutritional disorder in developed countries (2). Monosodium glutamate is a naturally occurring non-essential amino acid, but excessive consumption can lead to substantial metabolic alterations and severe bodily disorders (27). In neonatal rats, MSG treatment causes hypothalamic lesions, disrupting feeding behavior, increasing food intake, and elevating blood glucose levels (28). Additionally, MSG disrupts brain functions and induces oxidative stress, which may result in sudden neuronal death, acute degenerative changes, and chronic neurological defects (29).

This study found noteworthy increases in body weight, BMI, and Lee Index in the MSG-treated group in relation to controls. Neonatal MSG administration, as a model of obesity, damaged the hippocampus and led to the formation of neuronal artifacts during adolescence (PND50), visible as DNs in toluidine blue staining. The density of DNs in various hippocampal regions was noteworthyly higher in the MSG group than in the control group. Additionally, oxidative damage in the hippocampus was characterized by reduced activity of antioxidant enzymes (CAT, SOD, and GPx) and increased lipid peroxidation in MSG-treated rats. Notably, hippocampal AChE activity was noteworthyly elevated in the MSG group in relation to controls. However, treatment with Co-Q10 improved hippocampal antioxidant activity, inhibited AChE, and noteworthyly reduced DN density in various hippocampal subareas, preventing MSG-induced memory and learning impairments.

Glutamate crosses the BBB in limited quantities through active transport, which is tightly regulated to protect the brain from excitotoxicity. However, because the BBB is underdeveloped at birth, MSG can more easily penetrate the brain, reducing arcuate nucleus activity and neurons in the satiety center. As a result, neonatal MSG-treated rats often exhibit hyperphagia and obesity (30). Following MSG treatment, brain plasma glutamate concentrations rise substantially above normal levels, leading to excessive glutamate receptor activation and resulting in apoptosis and necrosis of neuronal cells (31). This overactivation releases calcium ions from intracellular stores and maximally activates mitochondria, as well as endonucleases, phospholipases, and proteases, which destroy cellular structures, plasma membranes, and DNA (32).

The hippocampus, a region particularly vulnerable to oxidative stress and glutamate-induced excitotoxicity, plays a crucial role in spatial learning and memory (33). Memory impairments associated with hippocampal dysfunction (working-spatial memory) monitored in the MSG group in this study may be due to alterations in glutamate neurotransmission and changes in glutamate receptor expression. Research has shown that loss of NMDA glutamate receptors in the hippocampus can impair learning (34). Furthermore, increased glutamate accumulation in the hippocampus has been linked to damage in CA₁ pyramidal neurons, as monitored in this study (35).

Acetylcholine is a critical neurotransmitter in the brain, and disruptions in its storage, release, and metabolism are associated with behavioral, cognitive, learning, and memory disorders, as well as neurodegenerative diseases (36). This study demonstrated that neonatal MSG administration increased hippocampal AChE activity. Increased AChE activity accelerates acetylcholine metabolism in the synaptic cleft and reduces cholinergic neurotransmission efficiency, causing progressive cognitive impairments (36). Furthermore, previous studies indicate that MSG induces oxidative stress by increasing lipid peroxides and ROS, which may further elevate AChE activity (37). In contrast, other research has found that L-glutamate administration decreased AChE activity (38). However, the dose used in those studies (103 mg/kg) was much lower than the acute dose (4 g/kg) used in the present study, and it was administered during adulthood rather than the neonatal period. In this study, Co-Q10 at 20 mg/kg inhibited AChE activity. Given that AChE inhibitors are the first-line treatment for cognitive disorders, dementia, and AD, Co-Q10, which has minimal side effects, could be a promising alternative to traditional drugs that are associated with undesirable side effects such as nausea, diarrhea, and appetite loss (39). Kumar et al. demonstrated that Co-Q10 improves cognitive functions and reduces AChE activity in AD models in adult rats (40).

Hippocampal oxidative stress, as indicated by increased lipid peroxidation and decreased activity of CAT, SOD, and GPx, is one of the toxic effects of MSG. Oxidative stress is a hallmark of neurological diseases. Despite the brain's high metabolic activity, it has limited antioxidant capacity. The production of free radicals leads to lipid peroxidation and oxidative DNA damage, resulting in cellular damage and apoptosis (41). Oxidative stress also disrupts BBB function, stimulates glutamate receptors, and activates inflammatory and apoptotic signaling pathways (42).

Conversely, this study demonstrated that Co-Q10 possesses potent antioxidant properties, improving working and avoidance memory in MSG-induced obese rat models. Coenzyme Q10 is most concentrated in tissues with high energy demands, such as the brain. It plays a vital role in cellular processes, including regulating cellular metabolism, oxidative stress, H₂O₂ formation, bioenergetics, and gene regulation (43). Coenzyme Q10 enhances mitochondrial function in neurons by increasing ATP synthesis and has demonstrated neuroprotective effects in various neurological and psychiatric disorders, including AD, PD, and MDD (44). Following neonatal MSG treatment, this study monitored an increased density of DNs in various hippocampal subareas. Rapid glutamate release can induce DN formation in the brain (45), which persists into adolescence. Dark neurons are dying or degenerating neurons characterized by a hyperbasophilic appearance and highly condensed ultrastructure. Dark neurons prevalence has been monitored in the CA₁, amygdala, cortical pyramidal layer, and other limbic structures in various brain injury models, as evidenced by cresyl violet or toluidine blue staining (46). Our study showed that the effects of MSG on CA_1_ and CA_2_ vulnerability are different. Although recent studies on the selective vulnerability of CA_1_ to CA_3_ neurons have shown conflicting results, and depending on several factors, such as the method and animal model, the vulnerability of different hippocampal subfields will vary. However, considering possible mechanisms (intrinsic oxidative stress potential and mitochondrial dysregulation) in CA_1_ and CA_2_ can explain this differential vulnerability (35).

### 5.1. Conclusions

The MSG-induced obesity model resulted in noteworthy damage to the hippocampus of rats, characterized by a high density of DNs. Reduced hippocampal antioxidant capacity and increased AChE activity also impaired working and avoidance memory. Furthermore, Co-Q10 noteworthyly ameliorated hippocampal cellular damage, reduced the density of DNs, and, through its antioxidant effects and AChE inhibitory action, improved memory and learning in the pathology induced by neonatal MSG consumption. Treatment with Co-Q10 offers a promising potential for the prevention and treatment of complications associated with MSG toxicity. At the end, we recommended the investigation of long-term effects of Co-Q10 on cognitive function, exploration of the potential gender differences, examination of the molecular pathways, particularly its impact on glutamate receptors and synaptic plasticity, evaluation of the efficacy Co-Q10 in combination with other antioxidant compounds for synergistic effects in preventing MSG-induced cognitive impairments and conduction of clinical trials to determine the applicability of Co-Q10 in preventing and treating cognitive impairments in humans exposed to high MSG levels.

## Data Availability

The dataset presented in the study is available on request from the corresponding author during submission or after publication. The data are not publicly available because the data of this study are taken from the Ph.D. thesis of the first author. The authors are advised not to send the data at this stage, if any of the referees or editors request to send specific data, the data will be sent.
